# Genetic alterations associated with ALTered telomeres

**DOI:** 10.18632/oncotarget.26111

**Published:** 2018-09-18

**Authors:** Jacqueline A. Brosnan-Cashman, Mindy K. Graham, Christopher M. Heaphy

**Affiliations:** Christopher M. Heaphy: Departments of Pathology and Oncology, The Johns Hopkins University School of Medicine, Baltimore, MD, USA

**Keywords:** alternative lengthening of telomeres, DAXX, ATRX, KIFC3, telomeres

Telomeres are comprised of double-stranded TTAGGG hexameric repeats located at the terminal ends of eukaryotic chromosomes and are pivotal for maintenance of genome integrity. When telomeres become critically short due to progressive telomere shortening following each round of cell division, normal cells undergo p53-dependent apoptosis or replicative senescence. In contrast, in order to maintain unlimited replicative capacity, cancer cells usually maintain telomere lengths by expressing the enzyme telomerase, a telomere-specific reverse transcriptase. However, a subset of all cancers maintains telomere lengths through a telomerase-independent telomere maintenance mechanism, termed Alternative Lengthening of Telomeres (ALT). Due to telomere deprotection and altered chromatin dynamics, telomere length maintenance in these ALT-positive cancers are maintained through a homology-directed DNA repair mechanism [1]. Classic features of ALT include dramatic telomere length heterogeneity, the presence of ultra-bright telomeric foci (often, but not always co-localized with the promyelocytic leukemia protein, PML), and circular extrachromosomal C-rich telomeric DNA (i.e. C-circles).

Our research group previously uncovered a strong correlation between ALT-positive cancers and somatic inactivating mutations in two chromatin remodeling genes, death domain associated protein (*DAXX*) and alpha-thalassemia/mental retardation X-linked (*ATRX*) [2]. Others have reported similar findings in multiple tumor types, including pancreatic neuroendocrine tumors (PanNETs), gliomas, neuroblastomas, and sarcomas (e.g. osteosarcoma) [3, 4]. The ATRX/DAXX complex deposits histone variant H3.3 in heterochromatic regions of chromosomes containing highly repetitive sequence elements, such as retrotransposons, pericentromeric regions, and telomeres. These repetitive sequences are inherently unstable and are prone to increased replication errors and aberrant homologous recombination. As a result, disruption of ATRX/DAXX chromatin remodeling function at telomeres presumably allows for the development of ALT.

The vast majority of ALT-positive cancers and cell lines have lost functional ATRX [5]. However, in ALT-positive PanNETs, DAXX alterations are twice as prevalent as ATRX alterations, and both frequently occur in conjunction with MEN1 inactivation [2, 3]. Multiple studies have demonstrated that ALT and ATRX/DAXX loss in primary PanNETs are independently associated with aggressive clinicopathologic behavior and reduced recurrence-free survival. Interestingly, DAXX alterations were recently found in a subset of Hürthle cell carcinomas, although whether these specific cases exhibited ALT is unknown [6]. In contrast to PanNETs, ALT-positive gliomas typically harbor ATRX alterations in the context of *IDH1/2* and *TP53* mutations. In addition, inactivating mutations in *SMARCAL1*, an ATP-dependent annealing helicase, have recently been identified to contribute to ALT in a subset of *ATRX*-wildtype, *TERT*-promoter-wildtype gliomas [7]. In fact, recent studies have also shown that two ALT-positive sarcoma cell lines, CAL78 and NY, do not express full length SMARCAL1 protein expression, but retain ATRX and DAXX protein expression, emphasizing the role of SMARCAL1 defects in ALT [7, 8].

Recently published work from Mason-Osann and colleagues has identified a novel mechanism of inactivating DAXX through chromosomal translocation [8]. The G292 osteosarcoma cell line has been previously determined to be ALT-positive, but did not contain alterations in ATRX [5, 9]. RNA-sequencing of G292 cells revealed a gene fusion event between the 3’ UTR of *DAXX* and the kinesin motor protein, *KIFC3*, leading to the translation of a chimeric DAXX-KIFC3 fusion protein. The nuclear localization of the DAXX protein was found to be unaffected by the fusion event, as was the association of the DAXX-KIFC3 chimera with ATRX and H3.3. However, the DAXX-KIFC3 fusion lacks DAXX exon 8, which encodes a SUMO interacting motif, thought to be critical for DAXX to localize to PML nuclear bodies. As such, the chimeric protein failed to colocalize with PML. Re-expression of wildtype DAXX in G292 not only allowed DAXX to form nuclear bodies with PML, but also reduced the levels of ALT-associated PML bodies, a key hallmark of the ALT mechanism. In summary, these findings are notable, as to our knowledge, this is the first *in vitro* evidence of altered DAXX function promoting ALT activity.

While the DAXX-KIFC3 fusion event was not identified in any additional cancers or cell lines, it highlights the breadth of possible genetic alterations that may give rise to ALT. In studies of ALT-suppressors in cancer, immunohistochemistry often reveals a minority of ALT-positive cases with intact nuclear expression of ATRX or DAXX. The identification of the DAXX-KIFC3 fusion illuminates the possibility of translocation events involving DAXX, or potentially other ALT-suppressors (e.g. ATRX or SMARCAL1), that may yield proteins with loss of function that are undetectable by immunohistochemistry in clinical specimens.

As proposed by the authors, we agree that comprehensive approaches combining next-generation sequencing (NGS), immunohistochemistry, and varied ALT detection methods (telomere-specific FISH, C-circle detection, and telomere reads from NGS) may help to further elucidate all of the genetic alterations contributing to ALT. Using these molecular techniques will more accurately identify ALT-positive tumors. This may be important for prognostication of tumor types or to identify unique molecular features of ALT-positive cancers. We, and others, have hypothesized that common ALT-associated molecular characteristics may be exploited therapeutically. For example, ALT-positive cancer cells may be more prone to inhibition of double-strand break responses and susceptible to ATR inhibition [9]. Additionally, ALT-positive cancer cells display elevated DNA damage at telomeres, thus strategies that induce replication stress (e.g. specific inhibitors of DNA polymerase delta, PCNA, or BLM) or more broadly via G-quadruplex stabilizing agents may hold therapeutic promise [4]. Enhanced detection of ALT-suppressors in cancer, along with an increased understanding of their mechanism(s) of action, will potentially allow for these ALT-specific precision medicine strategies to become a reality.
